# Data-Driven Techniques for Evaluating the Mechanical Strength and Raw Material Effects of Steel Fiber-Reinforced Concrete

**DOI:** 10.3390/ma15196928

**Published:** 2022-10-06

**Authors:** Mohammed Najeeb Al-Hashem, Muhammad Nasir Amin, Waqas Ahmad, Kaffayatullah Khan, Ayaz Ahmad, Saqib Ehsan, Qasem M. S. Al-Ahmad, Muhammad Ghulam Qadir

**Affiliations:** 1 Department of Civil and Environmental Engineering, College of Engineering, King Faisal University, Al-Ahsa 31982, Saudi Arabia; 2 Department of Civil Engineering, COMSATS University Islamabad, Abbottabad 22060, Pakistan; 3 MaREI Centre, Ryan Institute and School of Engineering, College of Science and Engineering, National University of Ireland Galway, H91 TK33 Galway, Ireland; 4 Department of Civil Engineering, NFC Institute of Engineering and Fertilizer Research, Faisalabad 38090, Pakistan; 5 Department of Environmental Sciences, Abbottabad Campus, COMSATS University Islamabad, Abbottabad 22060, Pakistan

**Keywords:** concrete, steel fibers, steel fiber-reinforced concrete, compressive strength, flexural strength

## Abstract

Estimating concrete properties using soft computing techniques has been shown to be a time and cost-efficient method in the construction industry. Thus, for the prediction of steel fiber-reinforced concrete (SFRC) strength under compressive and flexural loads, the current research employed advanced and effective soft computing techniques. In the current study, a single machine learning method known as multiple-layer perceptron neural network (MLPNN) and ensembled machine learning models known as MLPNN-adaptive boosting and MLPNN-bagging are used for this purpose. Water; cement; fine aggregate (FA); coarse aggregate (CA); super-plasticizer (SP); silica fume; and steel fiber volume percent (Vf SF), length (mm), and diameter were the factors considered (mm). This study also employed statistical analysis such as determination coefficient (R^2^), root mean square error (RMSE), and mean absolute error (MAE) to assess the performance of the algorithms. It was determined that the MLPNN-AdaBoost method is suitable for forecasting SFRC compressive and flexural strengths. The MLPNN technique’s higher R^2^, i.e., 0.94 and 0.95 for flexural and compressive strength, respectively, and lower error values result in more precision than other methods with lower R^2^ values. SHAP analysis demonstrated that the volume of cement and steel fibers have the greatest feature values for SFRC’s compressive and flexural strengths, respectively.

## 1. Introduction

The simple production method for concrete and the abundant availability of its ingredients and several applications make it the most widely used construction material around the globe. The nature of concrete is conventionally brittle, having low strain capacity, toughness, and energy absorption capability. Accordingly, researchers are searching for ways to minimize the brittleness of typical concrete by enhancing its tensile strength. The dispersed incorporation of short-discrete fibers in conventional cementitious concrete is emerging as an effective method of enhancing concretes’ capacity for energy absorption [1,2,3,4]. Multiple researchers have explored the addition of steel/synthetic/natural fibers to concrete as reinforcement for improving characteristics like fatigue resistance, toughness, ductility, and resist propagation of cracks in concrete [5,6,7,8,9,10,11,12,13,14,15,16]. Steel fibers are incorporated into concrete to enhance its post-cracking phenomenon and toughness [17,18,19,20]. SFRC have multiple applications in different sections of the construction industry like building, pavements, rehabilitation, and repair. The enhanced mechanical properties of SFRC, as reported by some of the researchers, for different applications are summarized in Table 1.

Currently, the practice adopted for evaluating the mechanical properties of SFRC is the performance of the entire experimental program. A considerable amount of time and cost is involved in determining an accurate connection between properties of material and mix design through experimentation [26]. The variable SFRC parameters are the aggregates, cement, water, admixture/super-plasticizer, additive material and fiber (i.e., steel fibers) contents, and the admixture type. Despite the considerable experimental research in the literature, it is hard to forecast the characteristics of SFRC with different mixtures with the help of computational approaches. Hence, the current work is focused on estimating SFRC mechanical characteristics by employing a soft computational approach.

The employment of soft computational techniques may assist in resolving multiple complex problems in various fields of engineering [27,28,29]. ML techniques may be applied to forecast the ultimate outcome after incorporating a database as input parameters. Two ML approaches, a single model-based standalone method and ensemble Bagging and AdaBoost models, are employed in this research for the estimation of SFRC properties. Per the reported studies, ensemble modelling techniques are more effective than an individual model as shown in Table 2. Chaabene et al. [30] reported a detailed assessment of applying ML techniques for predicting the mechanical properties of concrete. Furthermore, multiple types of research have been conducted to estimate the mechanical properties of different concrete types like self-healing concrete [31], high-performance concrete (HPC) [32,33,34,35,36], phase change materials-integrated concrete [37], and recycled aggregate concrete (RAC) [38,39,40,41]. Han et al. [33] employed machine learning techniques for estimation of HPC compressive strength. The input parameters included age, water, cement, coarse aggregates, sand, fly-ash, and GGBFS, and five variable combinations were considered. The accurate compressive strength of HPC was obtained by the developed model. In this study, the SFRC compressive strength is predicted by applying soft computational approaches. The current research will provide a base for conserving cost and time of future researchers.

ML techniques have demonstrated possible prediction results with least difference in trials for various concrete types. For said purpose, experimental methods, including casting and testing procedures, consume considerable cost, effort, and time. Therefore, the current need is to develop data modeling-based algorithms in line with closely linked in-dependent parameter identification and the instant decrement in input matrix dimensions. The application of soft computational approaches is gaining more importance for predicting concrete materials’ behavior in civil engineering. ML is an alternative technique for predicting SFRC’s compressive and flexural strengths to conserve experimental cost and time. The current study involves applying an individual ML model and multiple ensembled ML techniques to predict SFRC compressive and flexural strengths. In addition, the influence of raw materials on mechanical properties remains largely unexplored in contemporary study and is still very limited. The integration of SHapley Additive exPlanations (SHAP) with ML algorithms is also performed in this paper, addressing a current research need. SHAP analysis is intended to provide in-depth knowledge of SFRC mix design in terms of its mechanical strength factors via complicated nonlinear behavior and the description of input factors’ contributions by assigning a weighting factor to each input component. MLPNN is taken as an individual ML model, while MLPNN-AdaBoost and MLPNN-Bagging are taken as ensembled machine learning algorithms. In addition, statistical analysis is performed for the evaluation of all the considered models, and all said ML algorithms are compared as well. Afterwards, based on numerous statistical factors’ performance, a superior model is proposed for predicting SFRC properties. As a whole, a correlation for valuable structure properties is established in this research by applying interpretable machine learning techniques through feature importance.

## 2. Soft Computing Techniques

### 2.1. Multilayer Perceptron Neural Network (MLPNN)

**Figure 1:**
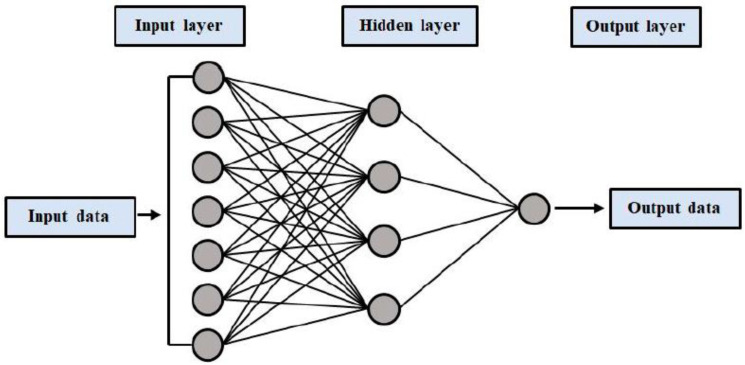
Architecture of a typical neural network [49].

The ANN model is among the most effective ML models. Its potential to resolve nonlinear issues has made it widely applied in hydrological and environmental engineering areas. Among multiple ANN models, the multilayer perceptron ANN (MLPNN) is the one that is used most frequently. The MLPNN model’s architecture comprises three layers: an input one followed by one or more hidden ones, and the output one. The three conventional functions of activation are; logsig, tansig, and purelin. Activations, weights, and bias functions are among the most important parts in both the output and hidden layers. The training of the model governs the parameters or weights of the model. The hidden layers employ the function of tansig activation; however, purelin is used for the output layer. The best structure is extracted by fivefold cross-validation. The top ANN model came out with three layers that are hidden (i.e., 9, 3, and 2) having optimal numbers of neurons against every hidden layer [48]. A typical/conventional neural network is shown in Figure 1. The composition of these networks is at three stages in a way that the input is transmitted by forward-pass, weight is multiplied by it, and the prediction of model output is made. The predicted results are then compared with the considered inputs. The input factors are considered for the model prediction outcomes. Based upon the objectives and performance, various loss functions are employed. The partial derivatives for cost function, linked with individual factors back in operation, are generated by backward propagation. Gradient descent is used for back loss propagation and model weight updating during this method.

### 2.2. MLPNN Bagging and MLPNN AdaBoost Techniques

The accuracy of prediction and recognition of ML can be improved by using ensemble approaches. These approaches usually help resolve problems by aggregating and integrating various algorithms having weaker predictions. A smart learner can be made by intelligently developing different sub-models (i.e., A, B, … N) along with the alteration of data for training and the merging of average and votes of combination measures, to obtain the correct result of projecting sub-models, for making an ideal model. The most frequently adopted ensembled modelling approach is Bagging, which implies the resampling bootstrap technique for calculating benefits and gathering the data. During this method, the first set of training with fractional algorithms is substituted for the actual algorithm. Some samples of data may seem in different algorithms, whereas few of them do not even appear in any model product. The average from all component models’ output is taken to calculate the final outcome model.

The Bagging approach, like boosting, creates a cumulative model that constructs multiple more precise components compared with non-ensemble models. Furthermore, the weighted averages are used in the Boosting process based on sub-models to determine their inclusion in the final model. Based upon MLPNN-like individual learners, the current work predicts the flexural and compressive strengths of SFRC using Bagging and Adaptive Boosting (AdaBoost) approaches.

## 3. Dataset

**Figure 2:**
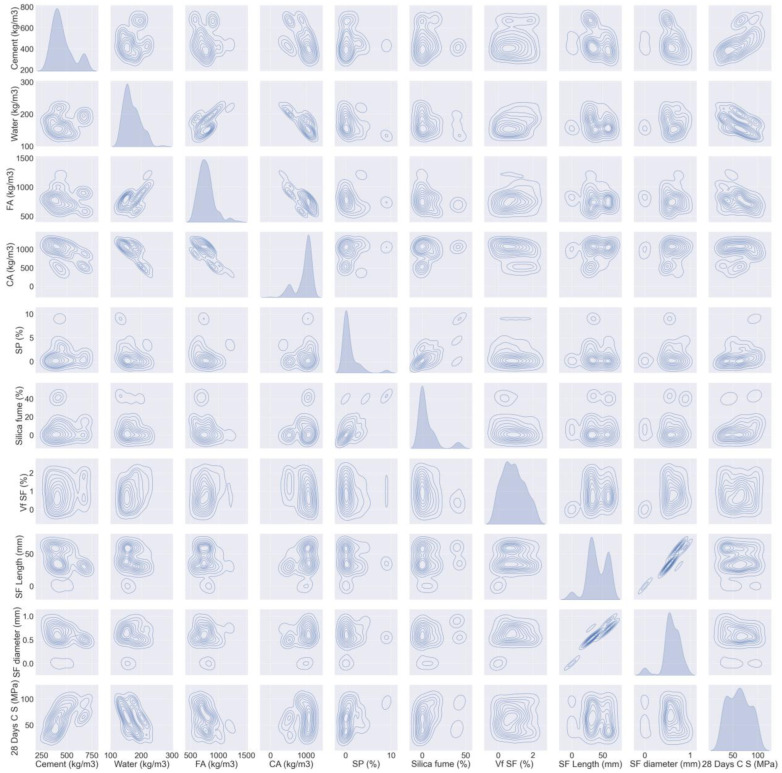
Data description of compressive parameters.

**Figure 3:**
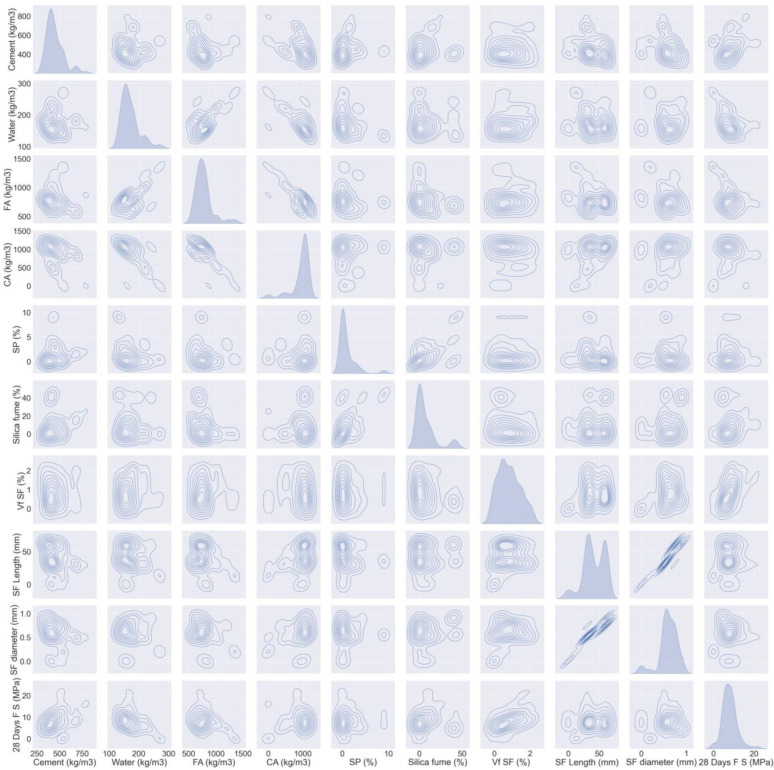
Data description of flexural parameters.

The literature-based dataset that was used comprises 150 mix ratios with 9 contribution parameters [50,51,52,53,54,55,56,57,58,59,60,61,62,63,64,65,66]. All the samples were water cured at 28 days. Figure 2 and Figure 3 exhibit the dataset that was employed to estimate SFRC strengths. These include water (kg/m^3^), cement (kg/m^3^), coarse aggregate (CA) (kg/m^3^), fine aggregate (FA) (kg/m^3^), superplasticizer (SP) (%), silica fume (%), volume fraction of steel fiber (Vf SF) (%), SF Length (mm), and SF diameter (mm). The prediction variables for output parameters (i.e., compressive and flexural strength) relied on the above-mentioned input parameters. Python scripting in Spyder Anaconda software was used to predict compressive and flexural strength.

## 4. Results and Discussion

### 4.1. Multiple-Layer Perceptron Neural Network (MLPNN)

**Figure 4:**
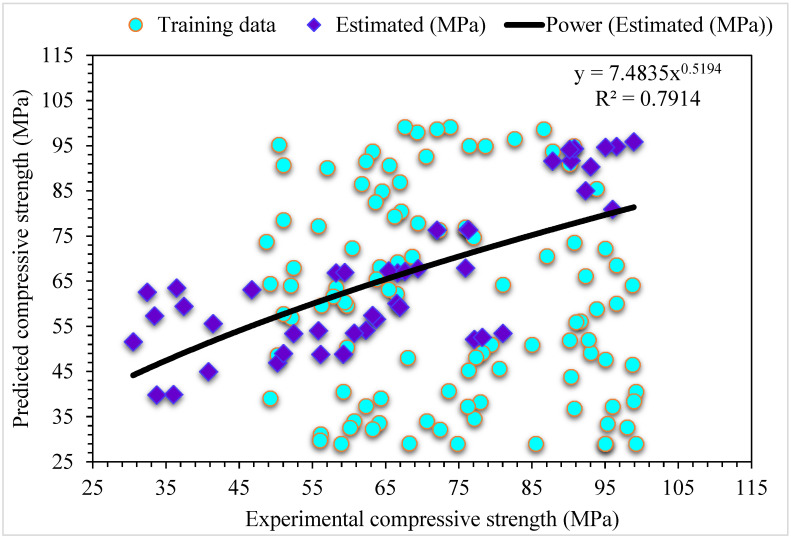
Experimental and MLPNN predicted results for compressive strength.

**Figure 5:**
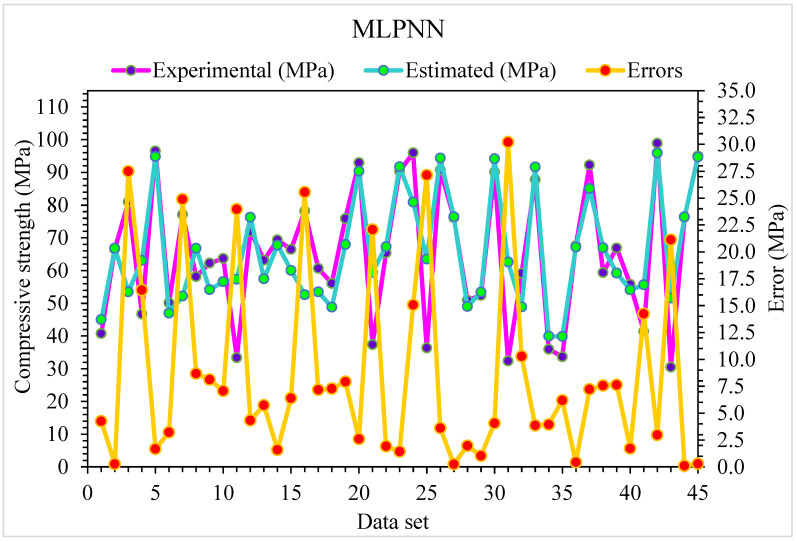
Experimental and MLPNN predicted values with errors for compressive strength.

The comparison of the MLPNN algorithm projected and experimental values for SFRC compressive strength are shown in Figure 4. MLPNN shows a reasonably estimated outcome with minimal variation in SFRC compressive strength. The appropriateness of the MLPNN model is represented by an acceptable R^2^ of 0.79. The error distribution of MLPNN predicted and experimental values for SFRC compressive strength are illustrated in Figure 5. The average error values for SFRC compressive strength are 8.69 MPa: 46% of the error values are below 5 MPa, 29% of these values range between 5 and 10 MPa, and 25% are more than 10 MPa.

**Figure 6:**
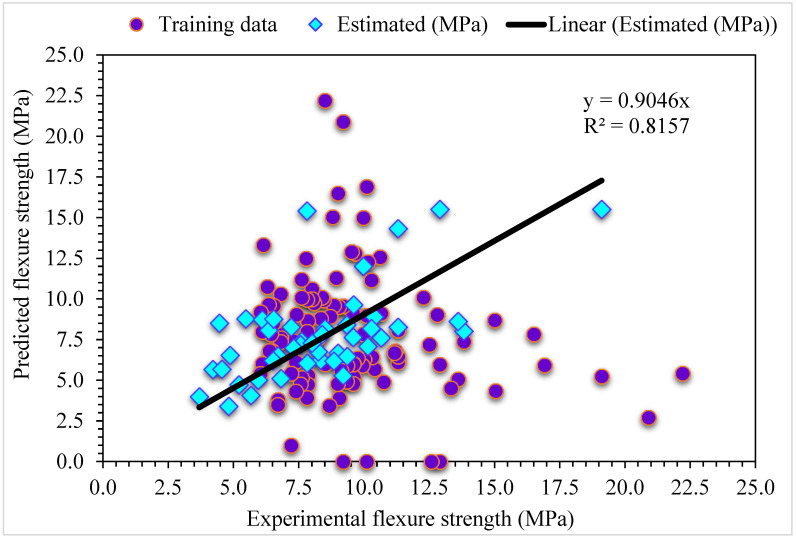
Experimental and MLPNN predicted results for flexural strength.

**Figure 7:**
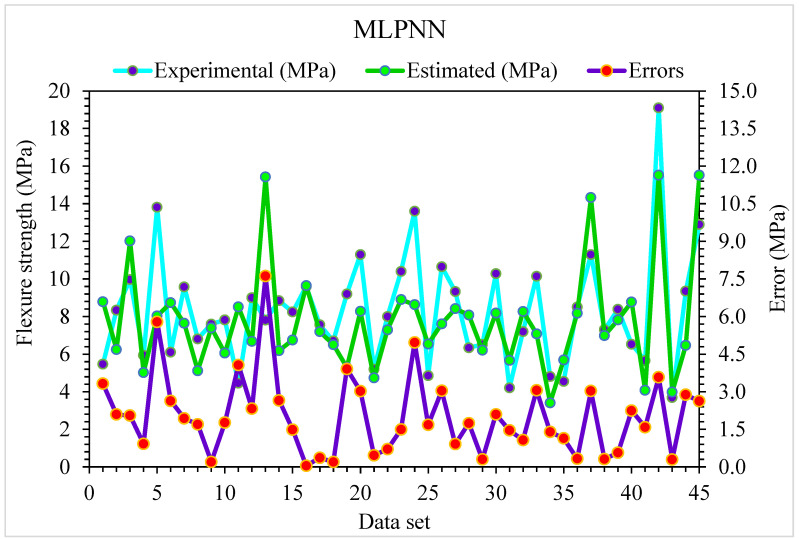
Experimental and MLPNN predicted values with errors for flexural strength.

The MLPNN projected and investigational results for SFRC flexural strength are presented in Figure 6. The R^2^ of 0.81 reveals the less appropriate outcome. Similarly, the projected results for the flexural strength of SFRC with the help of MLPNN lie in suitable array. The distribution of error for MLPNN projected, and investigational flexural strength of SFRC is shown in Figure 7. Nearly one third of values, 29%, are below 1 MPa, 66% are in the range of 1 to 5 MPa, and the remaining 5% are above 5 MPa.

### 4.2. MLPNN-AdaBoost

**Figure 8:**
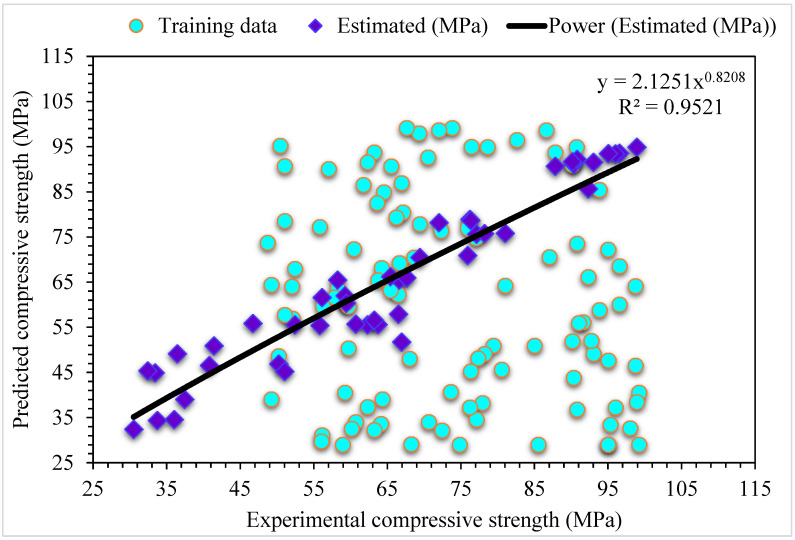
Experimental and MLPNN-AdaBoost predicted results for compressive strength.

**Figure 9:**
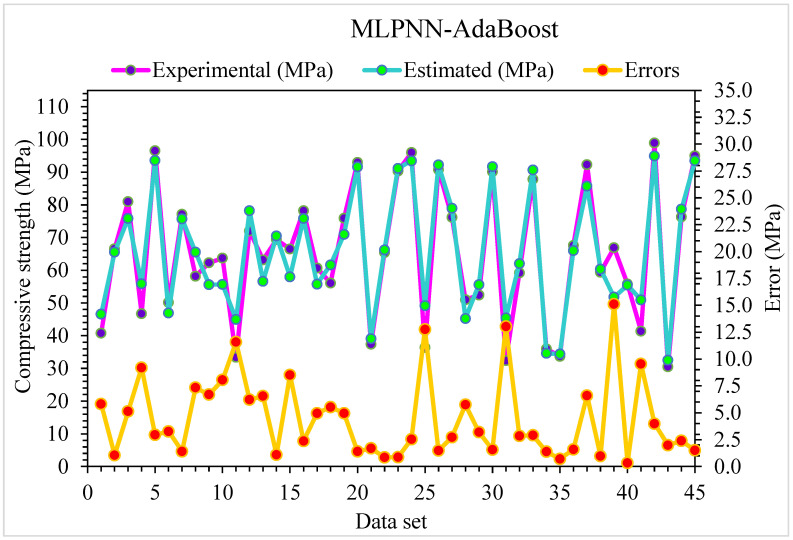
Experimental and MLPNN-AdaBoost predicted values with errors for compressive strength.

Figure 8 shows the predicted MLPNN-AdaBoost algorithm and investigational results for compressive strength of SFRC. The R^2^ of 0.95 for MLPNN-AdaBoost depicts a higher accuracy of outcomes than that of the MLPNN algorithm. Figure 9 represents the distribution of error for MLPNN-AdaBoost estimated and investigational results for the compressive strength of SFRC. It may be seen that 62% of values are below 5 MPa, 29% of these values range from 5 and 10 MPa, and 9% of values are above 10 MPa. The higher R^2^ and lower error values show more precision of the MLPNN-AdaBoost model than MLPNN.

**Figure 10:**
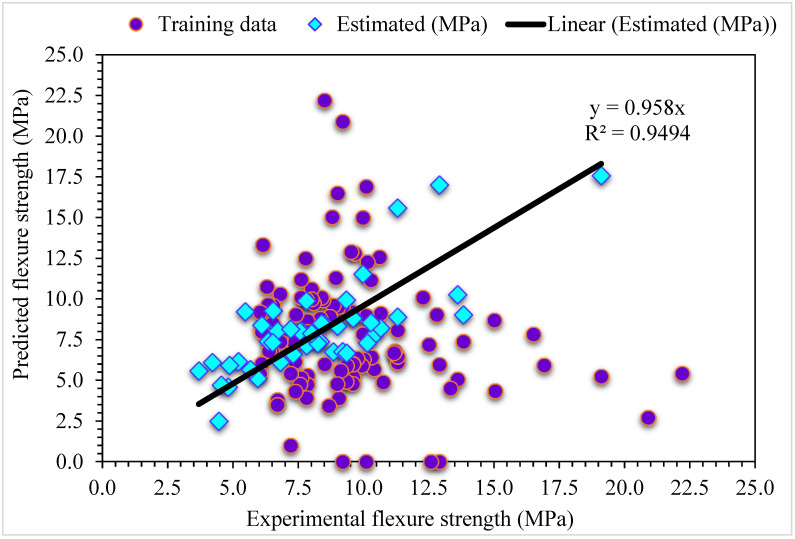
Experimental and MLPNN-AdaBoost predicted results for flexural strength.

**Figure 11:**
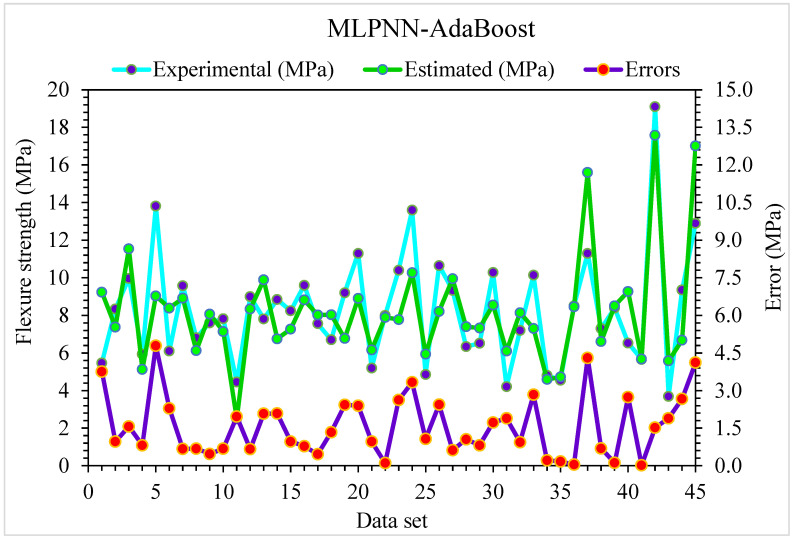
Experimental and MLPNN-AdaBoost predicted values with errors for flexural strength.

The MLPNN-AdaBoost model’s estimated and investigational results were compared for SFRC flexural strength (Figure 10). MLPNN-AdaBoost depicts reduced variation in error for SFRC flexural strength and highly precise predicted results. The adequacy of the MLPNN-AdaBoost model is represented by an acceptable R^2^ of 0.94. The error distribution of MLPNN-AdaBoost predicted and experimental SFRC flexural strength is illustrated in Figure 11. The average error value for SFRC flexural strength is 1.57 MPa: 47% of total error values are below 1 MPa, 53% of these values are between 1 and 5 MPa, and no value is more than 5 MPa.

### 4.3. MLPNN-Bagging

**Figure 12:**
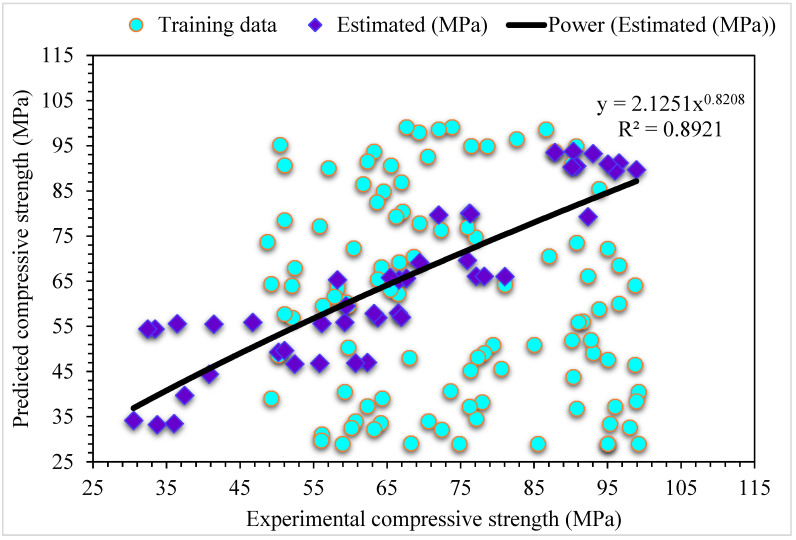
Experimental and MLPNN-Bagging predicted results for compressive strength.

**Figure 13:**
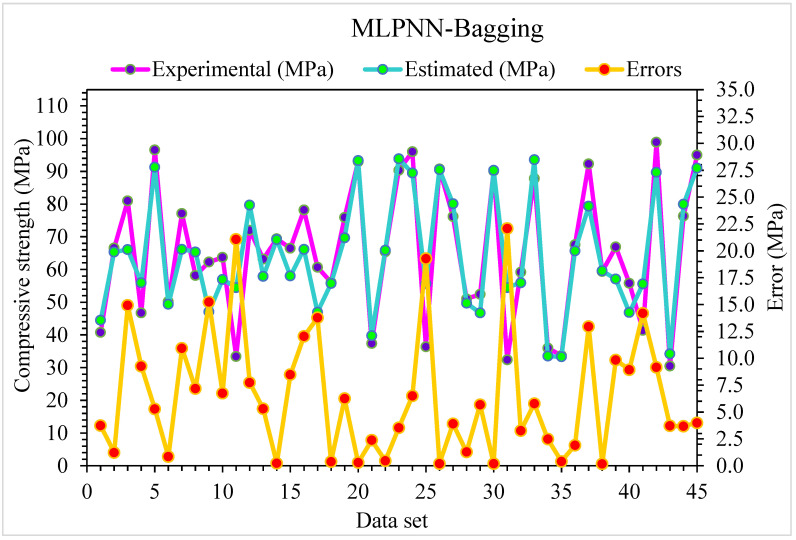
Distribution of experimental and MLPNN-Bagging predicted values with errors for compressive strength.

Figure 12 shows the projected and investigational outputs in case of MLPNN-Bagging for SFRC compressive strength. The R^2^ of 0.89 for this model depicts comparatively less appropriate results than the above-mentioned ensembled MLPNN-AdaBoost model. The estimated SFRC compressive strength outcomes for MLPNN-Bagging are superior to the individual MLPNN model. Figure 13 depicts the distribution of error for MLPNN-Bagging projected and investigational results for SFRC compressive strength: 49% of values are below 5 MPa, 28% are from 5 to 10 MPa, and the remaining 22% of these values are higher than 10 MPa. The R^2^ and error values for SFRC compressive strength in the case of MLPNN are more precise than the MLPNN-Bagging model. At the same time, the MLPNN ensembled machine learning algorithms error and R^2^ values are satisfactory. Therefore, this result shows the higher accuracy of estimation outcomes of MLPNN compared to other considered models.

**Figure 14:**
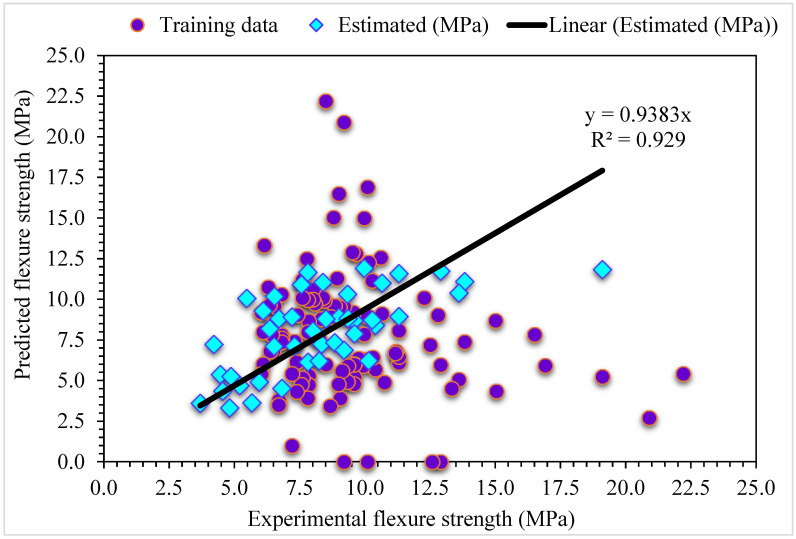
Experimental and MLPNN-Bagging predicted results for flexural strength.

**Figure 15:**
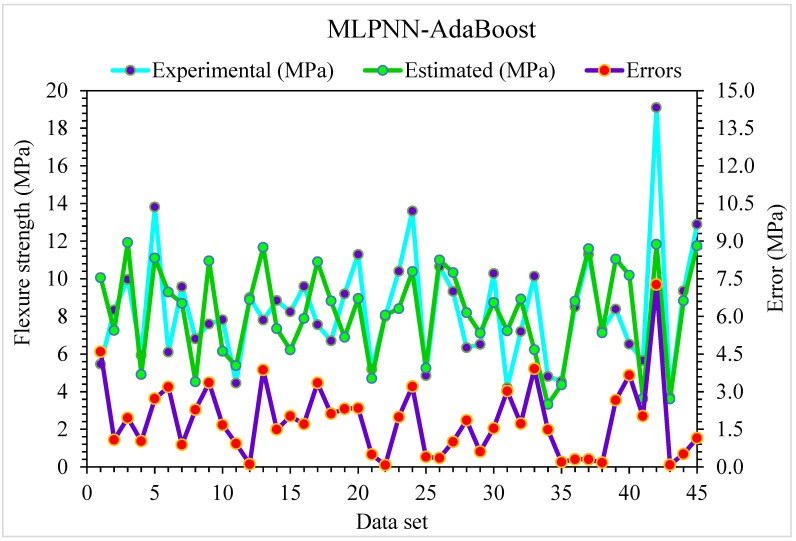
Distribution of experimental and MLPNN-Bagging predicted values with errors for flexural strength.

The estimated MLPNN-AdaBoost and investigational results for SFRC flexural strength are presented in Figure 14. The R^2^ of 0.92 for MLPNN-AdaBoost displays less accurate outcomes compared with MLPNN-AdaBoost. The distribution of error for the MLPNN-AdaBoost estimated and investigational results for SFRC flexural strength are presented in Figure 15. It is assessed that 33% of values are below 1 MPa, 62% lie in the 1 to 5 MPa range, and 4% are above 5 MPa. The higher R^2^ and lower error values demonstrate the higher accuracy of MLPNN-AdaBoost compared with MLPNN. In contrast, the attained R^2^ and error values for MLPNN-Bagging ensembled machine learning algorithms are suitable: this result showed the most accuracy for estimation outputs of MLPNN-AdaBoost compared with the other considered algorithms.

### 4.4. Comparison of All Models

**Figure 16:**
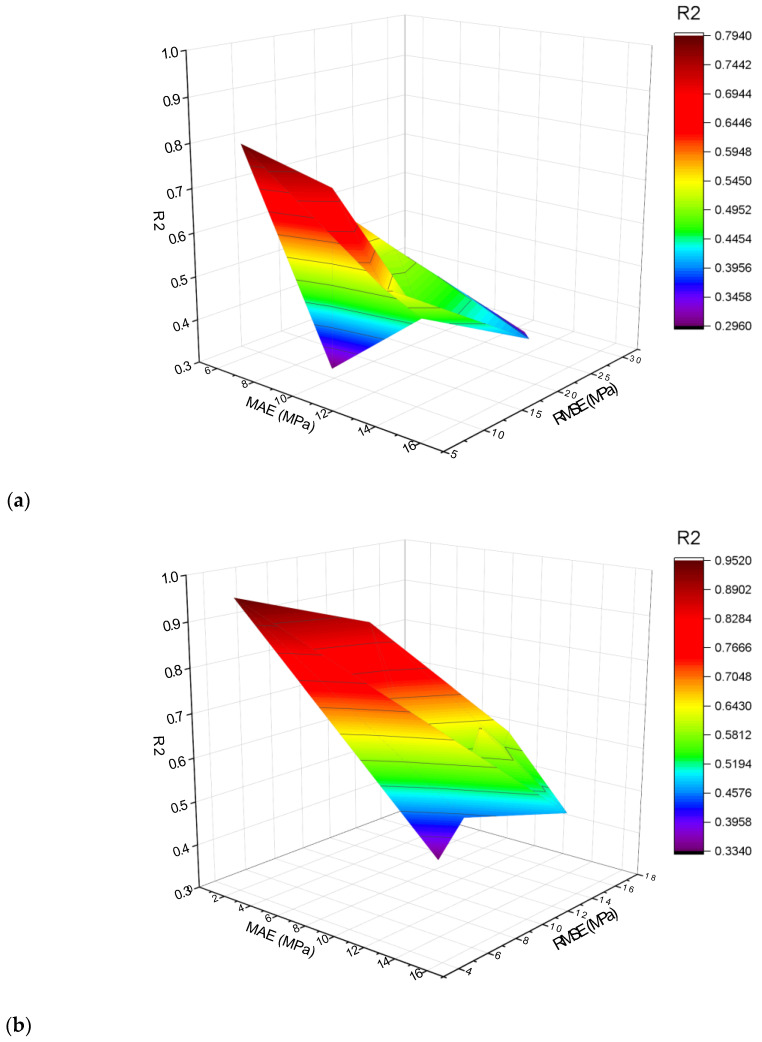

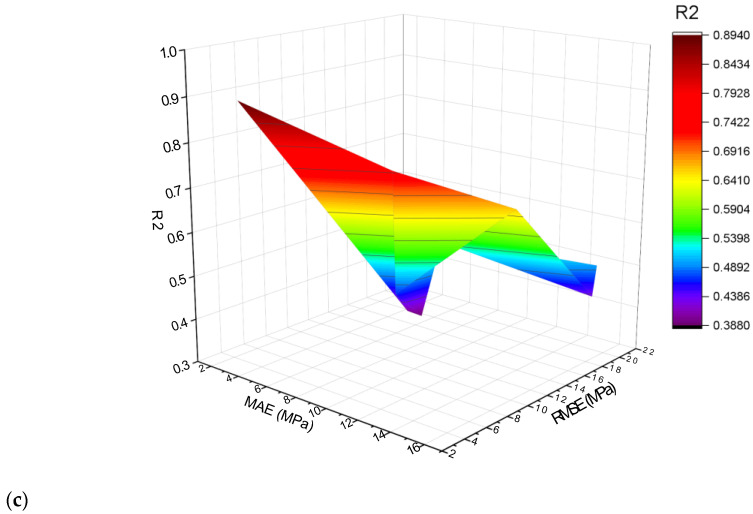
Compressive strength statistical representation: (**a**) MLPNN; (**b**) MLPNN-AdaBoost; (**c**) MLPNN-Bagging.

**Figure 17:**
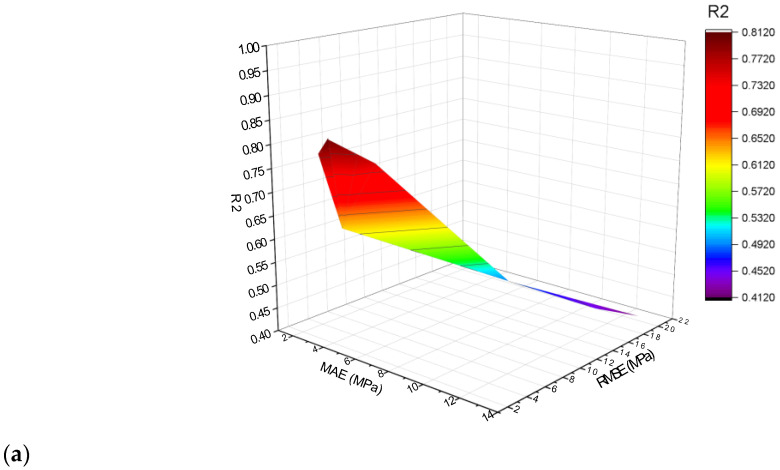

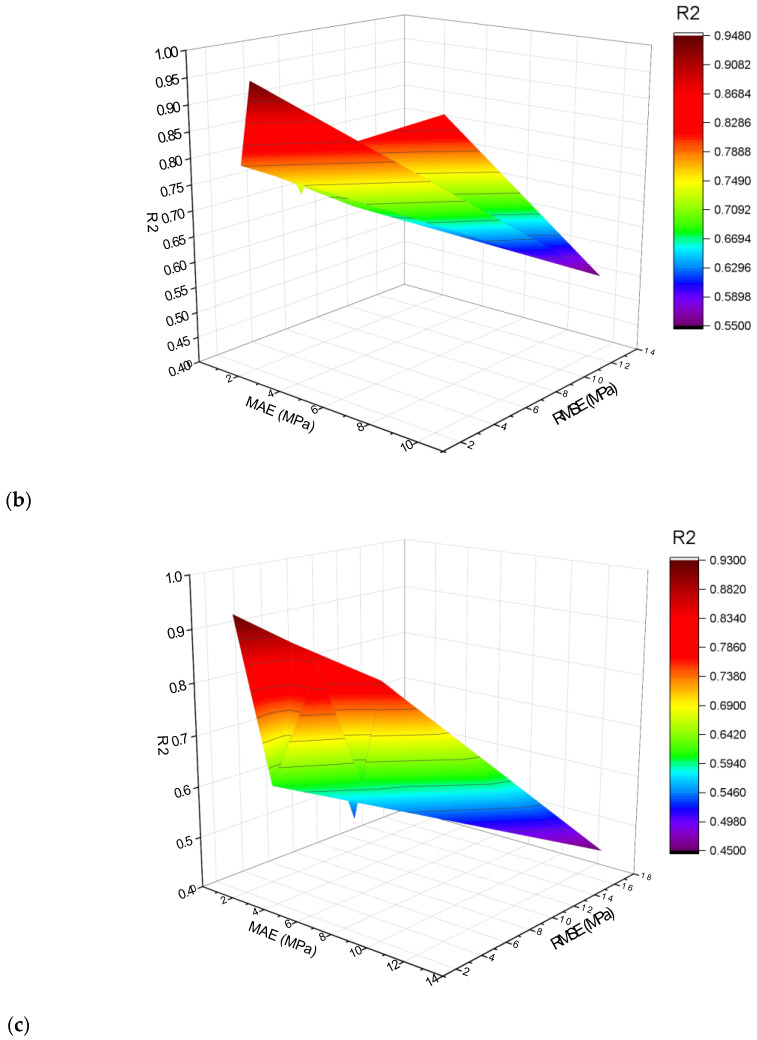
Flexural strength statistical representation: (**a**) MLPNN; (**b**) MLPNN-AdaBoost; (**c**) MLPNN-Bagging.

The k-fold technique was adopted for cross-validation in order to assess the performance of model while implementation. The performance of a model is determined by employment of statistical checks [67,68,69,70]. Normally, in the said k-fold process, there is splitting data in 10 clusters for random spreading by repeating this process 10 times for attaining suitable results. Table 3 provides the employed statistical checks. The compressive strength R^2^ values for MLPNN, MLPNN-Bagging, and MLPNN-AdaBoost models were 0.79, 0.89, and 0.95, respectively, as presented in Figure 16a–c. In the case of flexural strength, the R^2^ values for MLPNN, MLPNN-Bagging, and MLPNN-AdaBoost model were 0.81, 0.92, and 0.94, respectively, as presented in Figure 17a–c. It is observed that the R^2^ for MLPNN-AdaBoost is higher than those of the other considered algorithms, having lower error values for the SFRC compressive and flexural strengths.

**Figure 18:**
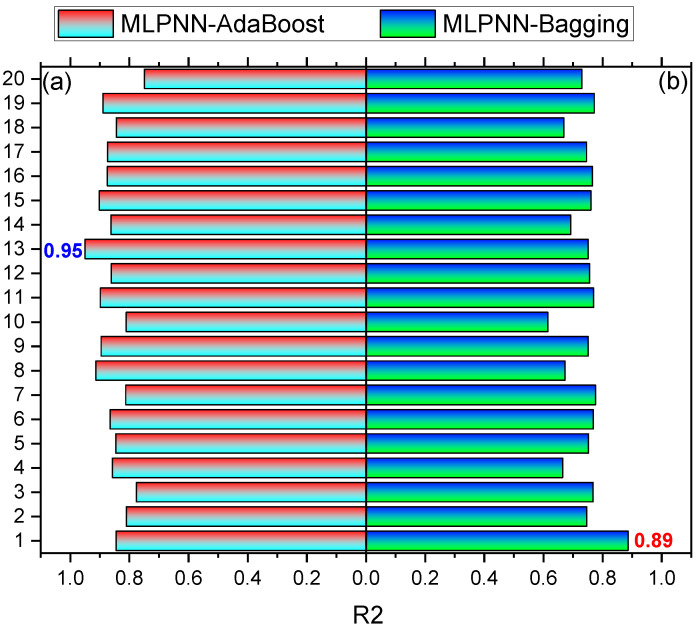
Compressive strength sub-models results: (**a**) MLPNN-AdaBoost; (**b**) MLPNN-Bagging.

**Figure 19:**
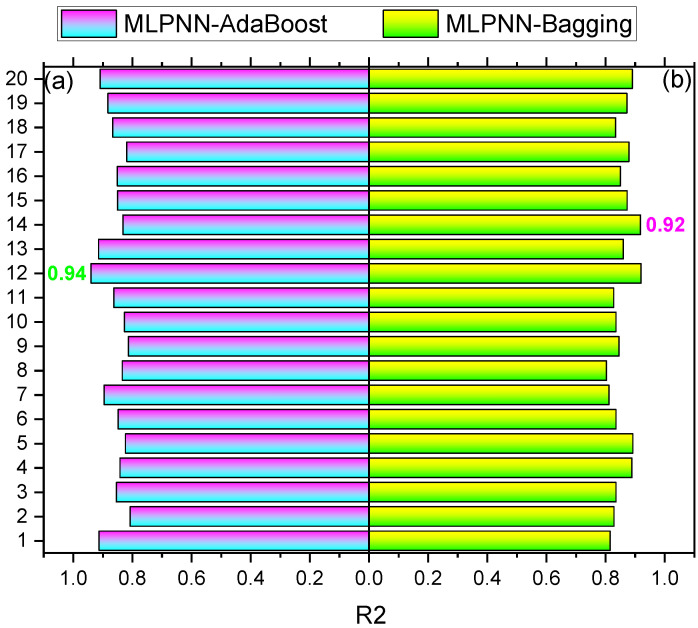
Flexural strength sub-models results: (**a**) MLPNN-AdaBoost; (**b**) MLPNN-Bagging.

The comparison of current models with the models in the literature is shown in Table 3. SFRC compressive strength is estimated by applying ensembled ML techniques in the current study, which intends to offer reliable and efficient results as compared to the other studies in the literature. The R^2^ of 0.95 for MLPNN-AdaBoost outcomes provides a more precise estimation of SFRC compressive strength. The ensembled MLPNN-AdaBoost ML models perform better in predicting compressive strength by utilizing an optimized model extracted from 20 sub-models, as presented in Figure 18a,b. It can be observed that ensembled MLPNN-AdaBoost models depict more accuracy and lower error than other models as well as the models reported in the literature. Despite this, SFRC flexural strength is estimated by applying ensembled ML techniques in the current study, which intends to offer reliable and efficient outcomes. The R^2^ of 0.94 in the case of MLPNN-AdaBoost results provides a more precise estimation for the compressive strength of SFRC. Out of 20 sub-models, an optimized model is used to estimate SFRC flexural strength in the case of ensembled MLPNN-AdaBoost ML models that perform better (Figure 19a,b). In comparison with other models, the ensembled MLPNN-AdaBoost models show higher accuracy and lower error.

## 5. Feature Importance of ML Models for Compressive and Flexural Strength

**Figure 20:**
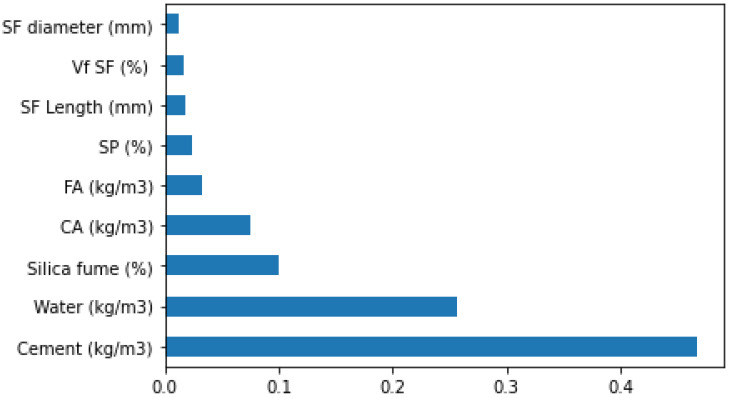
Compressive strength feature importance.

**Figure 21:**
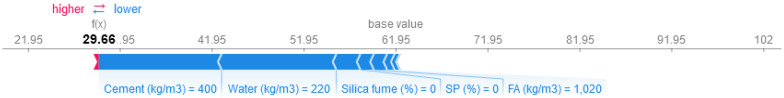
Compressive strength SHAP plot.

A thorough explanation is also given in the current research of the ML algorithm and interactions of considered input features. Different feature importance correlation for compressive strength of SFRC is shown in Figure 20. It can be observed that the feature value of cement feature is highest, i.e., 0.46, for SFRC compressive strength estimation. The cement feature has a positive influence, which means that by enhancing the cement content, the SFRC compressive strength increases. The SHAP plot (Figure 21) also shows that cement has the highest impact on SFRC compressive strength. The water feature has the second highest feature value of 0.26 for SFRC compressive strength; however, it influences negatively. Increasing the amount of water will reduce the compressive strength (Figure 21). Thirdly, the main factor for SFRC is silica fume, and this feature has approximately 0.1 feature value (Figure 20 and Figure 21). Further, the content of silica fume as a feature is positively influencing the SFRC compressive strength. It means that enhancement in its content turns into more compressive strength of SFRC. Coarse aggregate is next in line with a feature value of almost 0.8, but, in this case, the increase in coarse aggregate content up to optimum content only results in enhanced compressive strength. Beyond this optimum content of coarse aggregates, the SFRC compressive strength decreases. This behavior shows coarse aggregates’ positive and negative influence on SFRC compressive strength. Similarly, the feature value for sand, super-plasticizer is next, followed by steel fiber length, volume and diameter. All these considered features have more or less the same feature values near zero, showing their minimal impact on compressive strength of SFRC.

**Figure 22:**
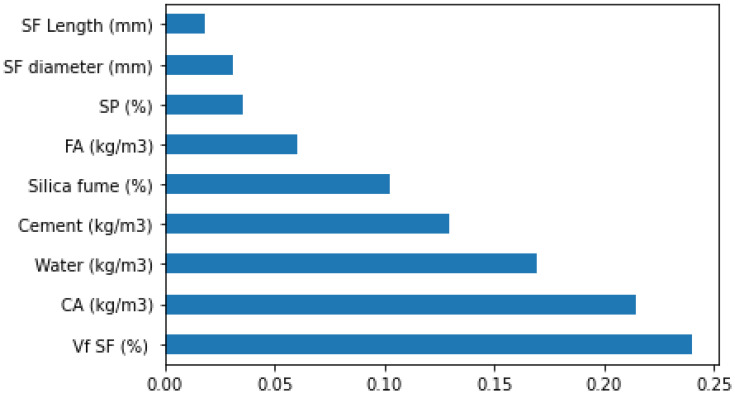
Flexural strength feature importance.

**Figure 23:**
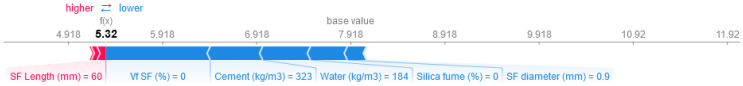
Flexural strength SHAP plot.

Similarly, Figure 22 and Figure 23 present the features’ importance correlations and features and SHAP plot for SFRC flexural strength. In this scenario, it is indicated in Figure 22 that the volume content of steel fiber has the highest feature value of 0.24 for flexural strength prediction of SFRC. It may also be observed from Figure 23 that the enhancing content of steel fiber volumes is increasing the SFRC flexural strength and vice versa. The second highest feature value of 0.22 is for coarse aggregates feature in the case of SFRC flexural strength. At the third level, the water has a feature value of 0.18, but with a negative influence, which means the enhancement in water content causes a reduction in flexural strength (Figure 23). Figure 22 depicts that the cement feature has a feature value up to 0.14 and positively influences the flexural strength of SFRC. The higher the cement content, the more the SFRC flexural strength (Figure 23). Afterwards, the silica fume, an important feature of SFRC, has a feature value of 0.11 for SFRC flexural strength, which is almost the same as for compressive strength of SFRC. The enhancing silica fume results in more SFRC flexural strength (Figure 23). Subsequently, the fine aggregates feature has a feature value of almost 0.07, followed by the feature values of super-plasticizer, steel fiber diameter, and length. The same feature values for all these features are nearly zero, depicting their lesser influence on SFRC flexural strength. The database used in the current study is the base of this prediction, and highly accurate results can be achieved with added data points.

This study assessed the compressive and flexural strength of 150 mixture proportions using 9 input factors with satisfactory performance. A substantially more accurate model might be generated by increasing the number of datasheets, importing a significantly larger number of mixes, and taking into account a greater number of input parameters. In order to increase the quantity of data points and outcomes in future research, it is recommended that experimental work, field testing, and numerical analysis utilizing a variety of techniques be employed (e.g., Monte Carlo simulation, among others). To improve the models’ performance, environmental conditions (such as high temperatures and humidity) might be incorporated in the input parameters along with a full explanation of the raw materials. The detailed limitations of machine learning models to estimate the strength properties of concrete is already reported in the literature [76].

## 6. Conclusions

The main aim of the current study is to determine the precision level of soft computational techniques to estimate SFRC compressive and flexural strengths. The considered input parameters for said prediction are cement, water, fine aggregate (FA), coarse aggregate (CA), super-plasticizer (SP), silica fume, the volume fraction of steel fiber (Vf SF), SF length (mm), and SF diameter (mm). The conclusions are as follows:

- As demonstrated by the R^2^ of 0.95, the MLPNN-AdaBoost technique may be applied for precise estimation of SFRC compressive strength from its actual dataset. In contrast, individual ML MLPNN and ensembled MLPNN-Bagging ML models have R^2^ values of 0.79 and 0.89, respectively, providing satisfactory results for SFRC compressive strength.
- The predicted compressive strength of SFRC is optimized by employing 20 sub-models from 10 to 200 estimators. SFRC compressive strength is more effectively predicted by an ensembled model MLPNN than other models. K-fold validation outcomes show that MLPNN models have lower MAE and RMSE with higher R^2^ for SFRC compressive strength than other considered models. The model for having the best prediction for SFRC compressive strength is MLPNN.
- Statistical checks like RMSE and MAE are used to evaluate the model’s performance. However, the superiority of MLPNN is demonstrated by its having a higher determination coefficient and fewer error values for SFRC compressive strength. The MLPNN is the most effective soft computational technique for predicting SFRC compressive strength.
- The cement content has the highest influence on compressive strength prediction of SFRC, followed by the contents of water, silica fume, and coarse aggregates, as revealed from SHAP analysis. The diameter of steel fibers has the least influence on SFRC compressive strength. The SHAP plot shows that the cement and silica fume content positively influence the compressive strength of SFRC.
- SFRC flexural strength is accurately predicted from its actual data by the MLPNN-AdaBoost technique as evident from the R^2^ of 0.94. However, the R^2^ of 0.81 and 0.92 in the case of individual MLPNN and ensembled MLPNN-Bagging ML models, respectively, estimated suitable results for SFRC flexural strength.
- The predicted flexural strength of SFRC is augmented by employing 20 sub models from 10 to 200 estimators. The more precise estimation of SFRC flexural strength is come out in case of an ensembled MLPNN model compared to other models. After applying the k-fold checks, the MLPNN algorithms are come out with higher R^2^ values and lower RMSE and MAE values for SFRC flexural strength than other models.
- MLPNN is come out with the best prediction for SFRC flexural strength. RMSE and MAE statistical checks are applied to evaluate the performance of the model. Similarly, the higher determination coefficient with lower values of error show the superiority of MLPNN for the prediction of SFRC flexural strength. Among soft computational techniques, MLPNN emerged as the most effective technique for the estimation of SFRC flexural strength.
- It is revealed from SHAP analysis that the volume of steel fiber significantly influenced the predicted SFRC flexural strength, followed by contents of coarse aggregates, water, cement, and silica fume. However, the SFRC flexural strength is least influenced by steel fiber length. The SHAP plot shows that the steel fiber volume positively influences the flexural strength of SFRC.

## Figures and Tables

**Table 1:** Reported applications and properties of SFRC.

Study Findings	Conducted Studies				
	Purkiss [21]	Patil and Sangle [22]	Noaman, et al. [23]	Boulekbache, et al. [24]	Gholamhoseini, et al. [25]
Studied Properties of SFRC	Residual Flexural Strength Residual Compressive Strength	Compressive Strength Flexural Strength	Compressive Strength Flexural Strength	Compressive Strength Flexural Strength	Compressive Strength Flexural Strength
Reported Outcomes	78–91% loss@800 °C 57–74% loss@800 °C	45.7 MPa 5.3 MPa	47 MPa 3.0–3.9 MPa	27–28.5 MPa 3.5–5.5 MPa	33.8–36.2 MPa 3.9–4.3 MPa
Considered Applications	Refractory Material	Beams	-	Structural Beams	Slabs

**Table 2:** ML techniques used in the previous studies.

Ref.	Material Type	Properties Predicted	ML Techniques Employed	No. of Input Parameters	Data Points	Best ML Technique Recommended
[42]	Concrete-Filled Steel Tubes	Ultimate axial capacity	Gene expression programming	6	227	-
[43]	Recycled aggregate concrete	Split-tensile strength	Gene expression programming, artificial neural network, and bagging regressor	9	166	Bagging regressor
[44]	Rice husk ash concrete	Compressive strength	Gene expression programming and random forest	6	192	Gene expression programming
[45]	Geopolymer concrete	Compressive strength	Decision tree, bagging regressor, and AdaBoost	9	154	Bagging regressor
[46]	Fly ash-based concrete	Compressive strength	Gene expression programming, artificial neural network, decision tree, and bagging regressor	7	98	Bagging regressor
[47]	Fly ash-based concrete	Compressive strength	Gene expression programming, decision tree, and bagging regressor	8	270	Bagging regressor

**Table 3:** Statistical checks of comparison of this study with the literature.

Material Type	Parameters	Techniques	MAE (MPa)	RMSE (MPa)	R^2^	References
SFRC	Compressive strength	MLPNN	8.7	12.3	0.79	This study
SFRC		MLPNN-AdaBoost	4.5	5.8	0.95	This study
SFRC		MLPNN-Bagging	6.6	8.8	0.89	This study
SFRC	Flexural strength	MLPNN	2.0	2.6	0.81	This study
SFRC		MLPNN-AdaBoost	1.6	2.0	0.94	This study
SFRC		MLPNN-Bagging	1.8	2.3	0.92	This study
Recycled coarse aggregate concrete (RCAC)	Compressive strength	SVM-AdaBoost	7.7	9.5	0.94	Amin, et al. [71]
Geopolymer concrete	Compressive strength	MLPNN	5.8	7.4	0.81	Amin, et al. [72]
Geopolymer concrete	Compressive strength	Support vector machine	6.7	8.1	0.78	Amin, et al. [72]
Waste marble powder Concrete (WMC)	Compressive strength	DT-AdaBoost	3.9	7.9	0.91	Khan, et al. [73]
Fly ash concrete	Compressive strength	Decision Tree	-	-	0.88	Khan, et al. [74]
Fly ash concrete	Compressive strength	MLP	-	-	0.90	Khan, et al. [74]
Fly ash concrete	Compressive strength	Bagging	-	-	0.93	Khan, et al. [74]
Geopolymer concrete	Compressive strength	Decision Tree	4.1	6.2	0.88	Zou, et al. [75]
Recycled coarse aggregate concrete (RCAC)	Compressive strength	DT-XGBoost	7.7	10.5	0.94	Amin, et al. [71]

## Data Availability

The data used in this research have been properly cited and reported in the main text.
